# Differential Expression of the Metabotropic P2Y Receptor Family in the Cortex Following Status Epilepticus and Neuroprotection *via* P2Y_1_ Antagonism in Mice

**DOI:** 10.3389/fphar.2019.01558

**Published:** 2020-01-16

**Authors:** Mariana Alves, Jonathon Smith, Tobias Engel

**Affiliations:** ^1^Department of Physiology and Medical Physics, Royal College of Surgeons in Ireland, Dublin, Ireland; ^2^FutureNeuro SFI Research Centre, Dublin, Ireland

**Keywords:** adenosine triphosphate, purinergic signaling, metabotropic P2 receptor family, status epilepticus, neurodegeneration, cortex

## Abstract

Purinergic signaling *via* P2 receptors is now widely accepted to play a critical role during increased states of hyperexcitability and seizure-induced pathology. In the setting of seizures and epilepsy, most attention has been paid to investigating the fast-acting ATP-gated P2X receptor family. More recent evidence has now also provided compelling evidence of an involvement of the slower-acting P2Y receptor family during seizures. This includes data demonstrating expression changes of P2Y receptors in the hippocampus following acute seizures and during epilepsy and anticonvulsive properties of P2Y-targeting drugs; in particular drugs targeting the P2Y_1_ subtype. Seizures, however, also involve damage to extra-hippocampal brain regions such as the cortex, which is thought to contribute to the epileptic phenotype. To analyze expressional changes of the P2Y receptor family in the cortex following status epilepticus and to determine the impact of drugs interfering with P2Y_1_ signaling on cortical damage, we used a unilateral mouse model of intraamygdala kainic acid-induced status epilepticus. Analysis of cortical tissue showed that status epilepticus leads to a global up-regulation of the P2Y receptor family in the cortex including P2Y_1_, P2Y_2_, P2Y_4_, and P2Y_6_, with the P2Y_1_ and P2Y_4_ receptor subtypes showing the strongest increase. Supporting a detrimental role of P2Y_1_ activation during status epilepticus, treatment with the P2Y_1_ agonist MRS2365 exacerbated high frequency high amplitude spiking, synonymous with injury-causing electrographic activity, and treatment with the P2Y_1_ antagonists MRS2500 protected against seizure-induced cortical damage. Suggesting P2Y_1_-mediated effects are predominantly due to increased microglia activation, treatment with the broad-spectrum anti-inflammatory drug minocycline abolished the observed neuroprotective effects of P2Y_1_ antagonism. In conclusion, our results further support a role for P2Y_1_-mediated signaling during seizure generation and seizure-induced neurodegeneration, suggesting P2Y_1_-targeting therapies as novel treatment for drug-refractory status epilepticus.

## Introduction

Epilepsy is characterized by an enduring predisposition to increased hyperexcitability states in the brain and is one of most common chronic brain diseases affecting up to 70 million people worldwide. Despite the increasing number of anti-epileptic drugs available in the clinic, drug resistance to pharmacological interventions remains steadily at 30% (Thijs et al., 2019). Status epilepticus, medical emergency defined as prolonged continuous seizure activity lasting longer than 5 min, is associated with high mortality and can cause wide-spread brain damage and serious neurological complications including the development of epilepsy (Betjemann and Lowenstein, 2015). As for epilepsy, drug refractoriness during status epilepticus remains equally high, with patients not responding to treatment being particularly vulnerable to adverse clinical outcomes (Novy et al., 2010). Mounting data has demonstrated an important role for neuroinflammation during both seizure generation and epileptogenesis (Vezzani et al., 2016); consequently, current research in epilepsy has a strong focus on the identification of the molecular mechanisms responsible for driving inflammatory processes during seizure-induced pathology.

Purinergic signaling *via* extracellular adenine and uracil nucleotide-activated P2 receptors has been suggested as possible link between neuroinflammation and increased hyperexcitability states (Henshall and Engel, 2015; Rassendren and Audinat, 2016; Alves et al., 2018). P2 receptors are subdivided into the fast-acting P2X receptor family, activated mainly by adenosine tri-phosphate (ATP) and consisting of seven members (P2X1-7) and the slower acting metabotropic P2Y receptor family, activated by ATP, adenosine di-phosphate (ADP) and the uracil nucleotides uracil tri-phosphate (UTP), uracil di-phosphate, and UTP-glucose consisting of eight members (P2Y_1,2,4,6,11,12,13,14_). P2Y receptors are further subdivided into groups based on their coupling to specific G proteins with P2Y_1_, P2Y_2_, P2Y_4_, P2Y_6_, and P2Y_11_ coupled to Gq proteins, ultimately resulting in the activation of protein kinase C *via* release of Ca^2+^ from intracellular stores. Among these, P2Y_11_ can also couple to Gs. P2Y_12_, P2Y_13_, and P2Y_14_ are coupled to Gi proteins decreasing cAMP production *via* inhibiting adenylate cyclase (von Kugelgen, 2006). Both P2X and P2Y receptors are distributed widely throughout the central nervous system where they are expressed and are functional on many cell types, including neurons, microglia, astrocytes, and oligodendrocytes (Burnstock, 2007).

Mounting data has repeatedly demonstrated distinct changes in the expression profile of P2X and P2Y family members following acute seizures and during epilepsy, and provided compelling evidence that drugs blocking P2X or P2Y members alter seizure severity and may even impact on the development of epilepsy (Amhaoul et al., 2016; Engel et al., 2016; Amorim et al., 2017; Alves et al., 2018). While most efforts have been invested to study the effects of the fast-acting P2X receptor family on seizures and epilepsy, in particular the P2X7 receptor (Beamer et al., 2017), increasing evidence also suggest a causal role for P2Y receptors during seizure-induced pathology (Eyo et al., 2014; Avignone et al., 2015; Alves et al., 2017; Alves et al., 2018; Alves et al., 2019). P2Y receptor expression is altered in the hippocampus following status epilepticus and during epilepsy (Alves et al., 2017) and ADP and UTP, both broad-spectrum P2Y receptor agonists, alter seizure severity during status epilepticus and seizure-induced neurodegeneration (Alves et al., 2017). Further supporting a role for P2Y receptors during seizures, mice deficient in P2Y_12_ display a more severe seizure phenotype during status epilepticus (Eyo et al., 2014). P2Y_1_ antagonism has been shown to reduce seizure severity and protect against hippocampal neurodegeneration (Simoes et al., 2018; Alves et al., 2019). This is of no surprise considering the well documented role of P2Y_1_ to regulate neurotransmitter release, facilitate neuronal excitability, and mediate microglia migration and activation (Guzman and Gerevich, 2016). Moreover, slice work in hippocampal tissue from epileptic rats showed that P2Y_1_ antagonism caused a reduction in astrocytic Ca^2+^-dependent glutamate gliotransmission and in turn hyperexcitability (Wellmann et al., 2018). Providing more evidence of an involvement of P2Y_1_ during seizure generation, P2Y_1_ antagonism decreases tumor necrosis factor-α–induced glutamate release from astrocytes and restores synaptic activity in hippocampal slices from epileptic mice (Nikolic et al., 2018).

To date, the investigation of P2Y expression and function has been mainly restricted to the hippocampus. Status epilepticus, however, also leads to cell death in extrahippocampal brain tissues including the amygdala, and piriform and entorhinal cortex in both experimental models of status epilepticus and in humans (Fujikawa et al., 2000; Curia et al., 2008; Mouri et al., 2008; Kienzler et al., 2009), which is thought to contribute to cognitive deficits and lowering of the seizure threshold (Thompson and Duncan, 2005; Helmstaedter, 2007). To establish whether P2Y signaling is involved in extrahippocampal neurodegeneration during status epilepticus, we characterized the expression profile of the P2Y receptor family in the cortex following status epilepticus and evaluated whether drugs targeting the P2Y_1_ receptor subtype protect the cortex from seizure-induced damage (Mouri et al., 2008).

## Materials and Methods

### Intraamygdala Kainic Acid Mouse Model of Status Epilepticus

All animal experiments were performed in accordance with the principles of the European Communities Council Directive (2010/63/EU). Procedures were reviewed and approved by the Research Ethics Committee of the Royal College of Surgeons in Ireland (REC 1322) and HPRA (AE19127/P038; AE19127/P001) and undertaken as described before (Engel et al., 2012). Experiments were carried out using 8- to 12-week-old C57Bl/6 male mice bred at the Biomedical Research Facility at RCSI and male *P2Y*_1_ *knock-out* (KO) mice obtained from The Jackson Laboratory (009131-B6.129P2-P2ry1 < tm1Bhk>/J). Animals were housed in a controlled biomedical facility on a 12-h light/dark cycle at 22 ± 1°C and humidity of 40% to 60% with food and water provided ad libitum. During stereotaxic procedures, mice were anesthetized using isoflurane (5% induction, 1%–2% maintenance) and maintained normothermic by means of a feedback-controlled heat blanket (Harved Apparatus Ltd, Kent, UK). Once fully anesthetized, mice were placed in a stereotaxic frame and a midline scalp incision was performed to expose the skull. A guide cannula (coordinates from Bregma; AP = −0.94 mm, L = −2.85 mm) and three electrodes for EEG recording (Bilaney Consultants, Sevenoaks, UK), two above each hippocampus and one above the frontal cortex as reference, were fixed in place with dental cement. EEG was recorded using the Xltek recording system (Optima Medical, Guildford, UK). Status epilepticus was induced by a microinjection of 0.3 µg kainic acid (KA) [0.2 µl phosphate-buffered saline (PBS)] (Sigma-Aldrich, Dublin, Ireland) into the right basolateral amygdala. Vehicle-injected control animals received 0.2 µl of PBS. The anticonvulsive lorazepam (6 mg/kg) (Wyetch, Taplow, UK) was delivered i.p. 40 min following intraamygdala KA or vehicle to curtail seizures and reduce morbidity and mortality.

### Drug Administration

Mice were assigned randomly to receive either vehicle (sterile H_2_O), P2Y_1_ antagonist MRS2500 (MRS25) (1 nmol) (≥96% purity; Tocris Bioscience, Abingdon, UK) or P2Y_1_ agonist MRS2365 (MRS23) (1 nmol) (98% purity; Tocris Bioscience, Abingdon, UK) 15 min following intraamygdala KA injection. All drugs were delivered by an intracerebroventricular (i.c.v.) microinjection (2 μl) into the ipsilateral lateral ventricle (coordinates from Bregma: AP = −0.4 mm; L = −0.95 mm). Minocycline (30 mg/kg, PBS) (Sigma-Aldrich (M9511), Dublin, Ireland) was administered twice *via* i.p. injection (200 μl) 24 and 4 h before triggering status epilepticus *via* intraamygdala KA (Alves et al., 2019).

### EEG Analysis

The duration of high-frequency (> 5 Hz) and high-amplitude (> 2 times baseline) polyspike discharges of ≥5 s duration, synonymous with injury-causing electrographic activity (Araki et al., 2002), was counted manually by a reviewer unaware of treatment as before (Engel et al., 2012).

### Western Blotting

To analyze expression changes of the P2Y receptor family in cortical tissue post-status epilepticus, the entire ipsilateral cortex was removed and homogenized in lysis buffer, and 30 µg of protein samples were loaded into an acrylamide gel and separated by SDS-PAGE electrophoresis. Membranes were probed with antibodies against P2Y_1_, P2Y_2_, P2Y_4_, P2Y_6_, P2Y_12_, P2Y_13_, and P2Y_14_ (Alomone Labs, Hadassah Ein Kerem, Jerusalem, Israel), c-Fos (Santa Cruz, Heidelberg, Germany), and β-actin (Sigma-Aldrich, Dublin, Ireland). Protein bands were visualized using a Fujifilm LAS-4000 system (Fujifilm, Tokyo, Japan) with chemiluminescence (Pierre Biotechnology, Rockford, IL, U.S.A.), which was followed by analysis using Alpha-EaseFC4.0 software.

### Fluoro-Jade B

Status epilepticus-induced neuronal cell death was assessed by Fluoro-Jade B (FjB) as described before (Engel et al., 2012). Twelve-micrometer coronal sections at the medial level of the hippocampus (Bregma AP = −1.94 mm) were sliced on a cryostat. Brain tissue was then fixed in 4% paraformaldehyde (PFA), rehydrated in ethanol, and transferred to a 0.006% potassium permanganate solution. Tissue sections were incubated with 0.001% FjB (Chemicon Europe Ltd, Chandlers Ford, UK) and mounted in dibutylphthalate polystyrene xylene mounting solution. Using an epifluorescence microscope, FjB-positive cells were counted under a 40× lens in two adjacent sections and the average determined for each animal.

### Immunofluorescence Staining

To perform immunofluorescence staining, mice were anaesthetized with an overdose of 250 µl sodium pentobarbital (200 mg/ml) delivered i.p. and transcardially perfused with 4% PFA. Brains were then transferred to a solution of PBS and immersed into a 4% agarose solution before sectioning in a VT1000S vibratome. Thirty-micrometer brain sections were incubated in 0.1% Triton X-100 and glycine followed by the blocking solution (1% BSA-PBS). Brain tissue was then incubated with the primary antibodies: P2Y_1_ (1:100) (Santa Cruz, Heidelberg, Germany), NeuN (1:400) (Millipore, Billerica, MA, U.S.A), GFAP (1:400) (Sigma-Aldrich, Dublin, Ireland), S100β (1:400) (Synaptic Systems, Goettingen, Germany), or Iba1 (1:400) (Wako, Neuss, Germany). Brain tissue was washed and incubated with a secondary antibody raised in goat conjugated with Alexa Fluor 488 and Alexa Fluor 568. Sections were stained with DAPI (1:500) and mounted onto glass slides with FluoroSave reagent. Confocal images were taken on a Zeiss Examiner Z1 microscope using a 40× immersion oil objective (Leica Microsystems, Wetzlar, Germany). Each image depicted in the results section is a representative picture from at least three mice. To determine the total number of P2Y_1_-positive NeuN and Iba1 cells, three images from the cortical layer V–VI were obtained using a 40× lens in the Zeiss Examiner Z1 confocal microscope. Cell counts were the result of the average counting of images and were carried out unaware of treatment groups.

### Statistical Analysis

For statistical analysis we used GraphPad Prism and STATVIEW software. Data was presented as means ± standard error of the mean. One-way analysis of variance with *post hoc* Fisher's protected least significant difference test was used to analyze three or more group data. For two-group comparison, Student's t-test was used to determine statistical differences between groups. Significance was accepted at *p < 0.05, **p < 0.01, and ***p < 0.001.

## Results

### P2Y Expression Changes in the Cortex Following Status Epilepticus

**Figure 1:**
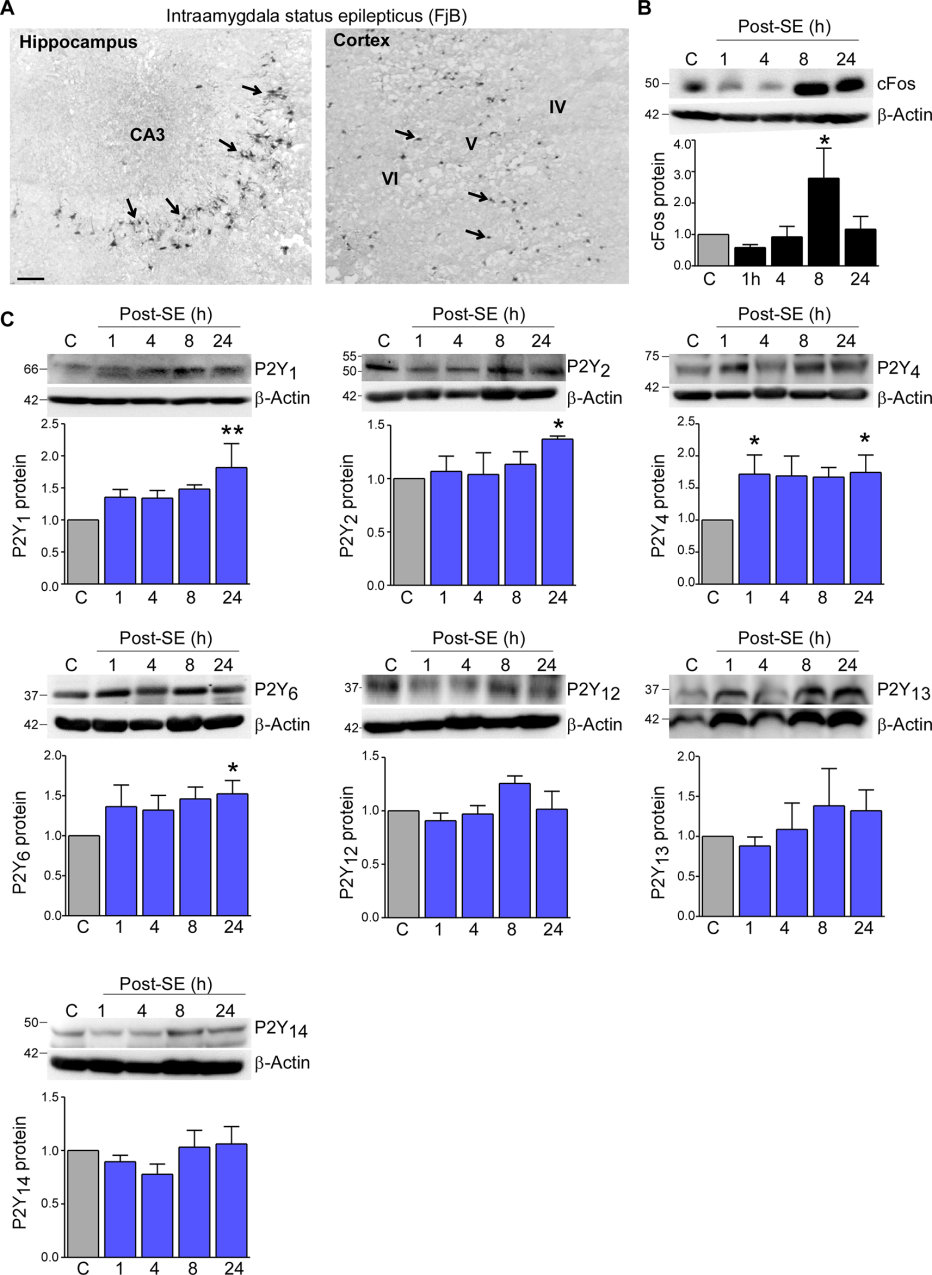
Expression profiling of the P2Y receptor family in the cortex following status epilepticus. **(A)** Photomicrograph (20× lens) showing neuronal damage 24 h following intraamygdala KA-induced status epilepticus in the ipsilateral hippocampus and cortex. Scale bar = 100 μm. **(B)** Representative Western blot (n = 1 per lane) and corresponding graph showing increased c-Fos expression in the ipsilateral cortex post-status epilepticus (n = 4 per group). **(C)** Representative Western blots (n = 1 per lane) and corresponding graphs showing the expression of the different P2Y receptor family members P2Y_1_, P2Y_2_, P2Y_4_, P2Y_6_, P2Y_12_, P2Y_13_, and P2Y_14_ in cortical tissue following status epilepticus. Of note, while P2Y_1_, P2Y_2_, P2Y_4_, P2Y_6_ are significantly increased post-status epilepticus, no changes could be observed for the remaining P2Y receptors (n = 6 per group). *p < 0.05, **p < 0.01.

To determine status epilepticus-induced changes in the expression profile of the P2Y receptor family in cortical tissue, we used the intraamygdala KA mouse model of status epilepticus (Araki et al., 2002; Mouri et al., 2008). In this model, status epilepticus leads to a characteristic lesion restricted to the ipsilateral brain hemisphere including the hippocampus and the cortex. While in the hippocampus cell death is mainly observed in the CA3 subfield, within cortical structures, neurodegeneration is most prominent in the cortical layers V and VI (**Figure 1A**) (Mouri et al., 2008). Increased levels of the activity-regulated protein c-Fos at 8 h following status epilepticus confirmed the recruitment of the ipsilateral cortex during status epilepticus (**Figure 1B**).

Previous work by us using the intraamygdala KA mouse model of status epilepticus has shown a distinct expression profile of the P2Y receptor family following status epilepticus (Alves et al., 2017). To determine whether status epilepticus also impacts on the expression of the P2Y receptor family in the cortex, tissue from the ipsilateral cortex was analyzed at different time-points post-status epilepticus *via* Western blot. This revealed an increase in the expression of several P2Y receptor family members including P2Y_1_, P2Y_2_, P2Y_4_, and P2Y_6_ (**Figure 1C**). While P2Y_1_, P2Y_2_, and P2Y_6_ showed a significant increase at 24 h post-status epilepticus, P2Y_4_ expression was already increased 1 h following status epilepticus and remained increased for up to 24 h (**Figure 1C**). Although displaying a slight increase in their expression, no significant expression changes could be observed for the remaining P2Y receptors post-status epilepticus (**Figure 1C**). P2Y_11_ expression was not analyzed due to the lack of a *P2ry_11_* gene ortholog in the mouse genome (Dreisig and Kornum, 2016).

Taken together, our results demonstrate changes in the expression of P2Y receptors in the cortex following status epilepticus with P2Y upregulation being the predominant response.

### Increased P2Y_1_ Expression in Microglia Following Status Epilepticus

Emerging evidence suggests a causative role for P2Y signaling during seizure generation and seizure-induced pathology (Alves et al., 2018). Among the P2Y receptors analyzed, P2Y_1_ was one of the receptors showing the strongest increase in its expression in the cortex following status epilepticus. Importantly, recent data suggests a functional role of P2Y_1_ during status epilepticus and epilepsy (Alves et al., 2019). Therefore, to test whether P2Y receptor activation impacts on cortical neurodegeneration, our subsequent studies focused on the P2Y_1_ receptor subtype.

**Figure 2:**
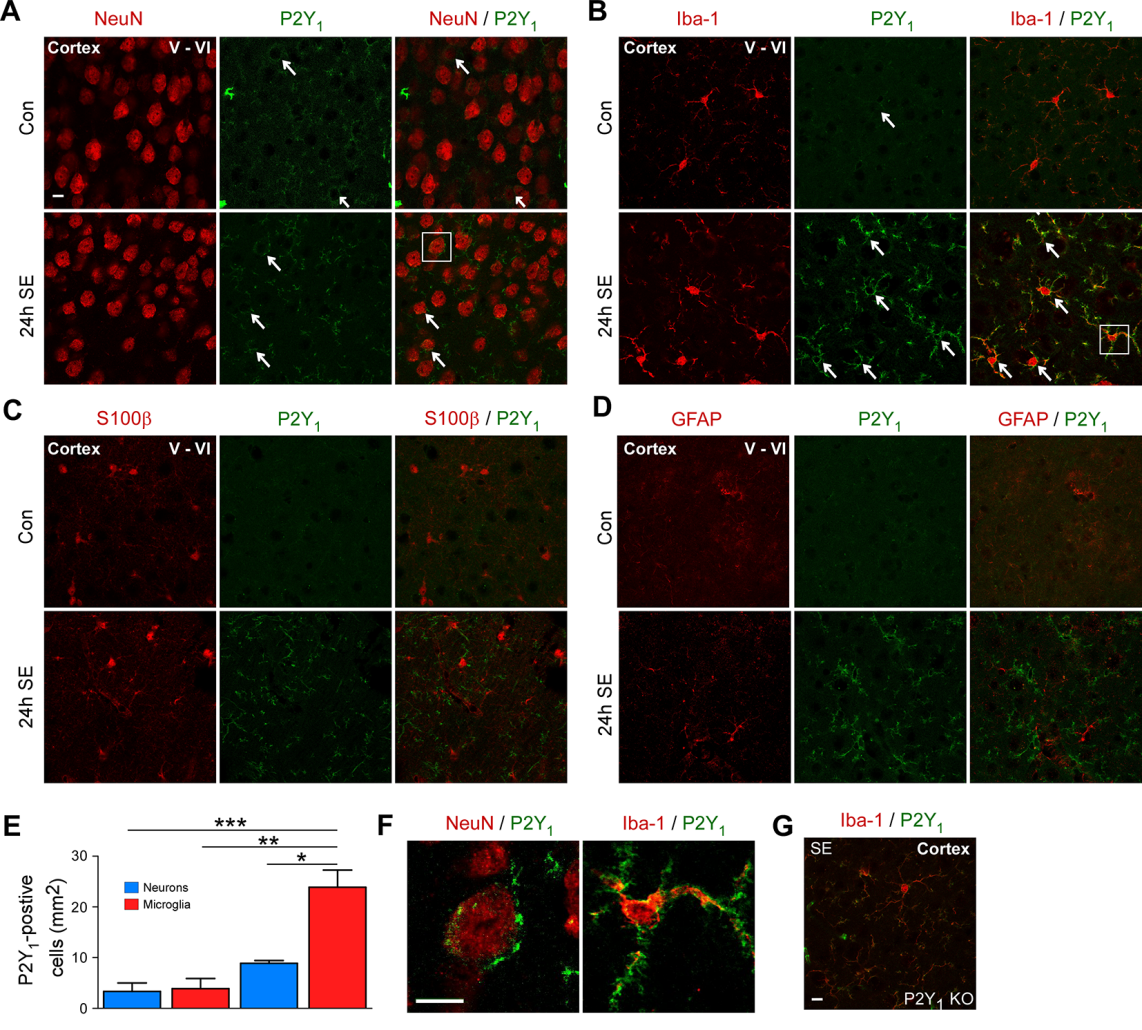
Cell-specific expression of P2Y_1_ post-status epilepticus. **(A)** Photomicrographs (40× lens) showing co-localization of P2Y_1_ (green) with neuronal marker NeuN in cortical tissue (layer V–VI) in control injected-vehicle mice and following status epilepticus (SE) (white arrows). Scale bar = 10 µm **(B)** Co-localization of P2Y_1_ (green) with microglia marker Iba1 (red) 24 h following status epilepticus (SE) in the cortex (white arrows). Scale bar = 10 μm. **(C, D)** No co-localization of P2Y_1_ (green) with the astrocyte markers GFAP and S100β (red). Scale bar = 10 μm. **(E)** Graph showing strong increase in P2Y_1_-positive microglia 24 h following status epilepticus (n = 3 per group). **(F)** Enlarged images, outlined by white box, showing co-localization of P2Y_1_ (green) on neurons (red) and microglia (red) 24 h post-status epilepticus (SE). Scale bar = 10 μm. **(G)** Specificity of P2Y_1_-detecting antibody was confirmed using tissue from P2Y_1_ knockout mice (KO) 8 h post-status epilepticus. Scale bar = 10 μm. Images are representative image from three animals per experiment. *p < 0.05, **p < 0.01, ***p < 0.001.

First, to explore the cell-specific expression pattern of P2Y_1_ in the cortex post-status epilepticus, we carried out co-immunostainings using different cell-type markers including NeuN for neurons, Iba1 for microglia, and GFAP and S100β for astrocytes and analyzed cortical layer V and VI, the areas were we observed most neuronal damage post-status epilepticus. Cortical tissue was analyzed 24 h following status epilepticus, the peak of P2Y_1_ post-status epilepticus expression in the cortex. While P2Y_1_ was detectable at low levels in cortical neurons and microglia under control conditions and following status epilepticus (**Figures 2A, B**), no co-localization was observed using the astrocyte markers GFAP and S100β in vehicle-injected control mice and in mice subjected to status epilepticus (**Figures 2C, D**). In line with previous results analyzing hippocampal P2Y_1_ expression (Alves et al., 2019), P2Y_1_ staining, however, strongly increased on Iba1-positive microglia 24 h post status epilepticus (**Figure 2E**). Higher magnification showed that, whereas cortical neuronal P2Y_1_ seemed to be localized to the cell body with a punctate expression pattern, microglia P2Y_1_ expression was observed throughout the cell including microglia processes (**Figure 2F**). Specificity of P2Y_1_ staining was confirmed using brain tissue from P2Y_1_ knock-out mice subjected to intraamygdala KA-induced status epilepticus (**Figure 2G**).

Thus, as observed previously in the hippocampus, status epilepticus leads to a strong increase in P2Y_1_ immunoreactivity on microglia in the cortex.

### P2Y_1_ Antagonism Decreases High Frequency High Amplitude Spiking During Status Epilepticus

**Figure 3:**
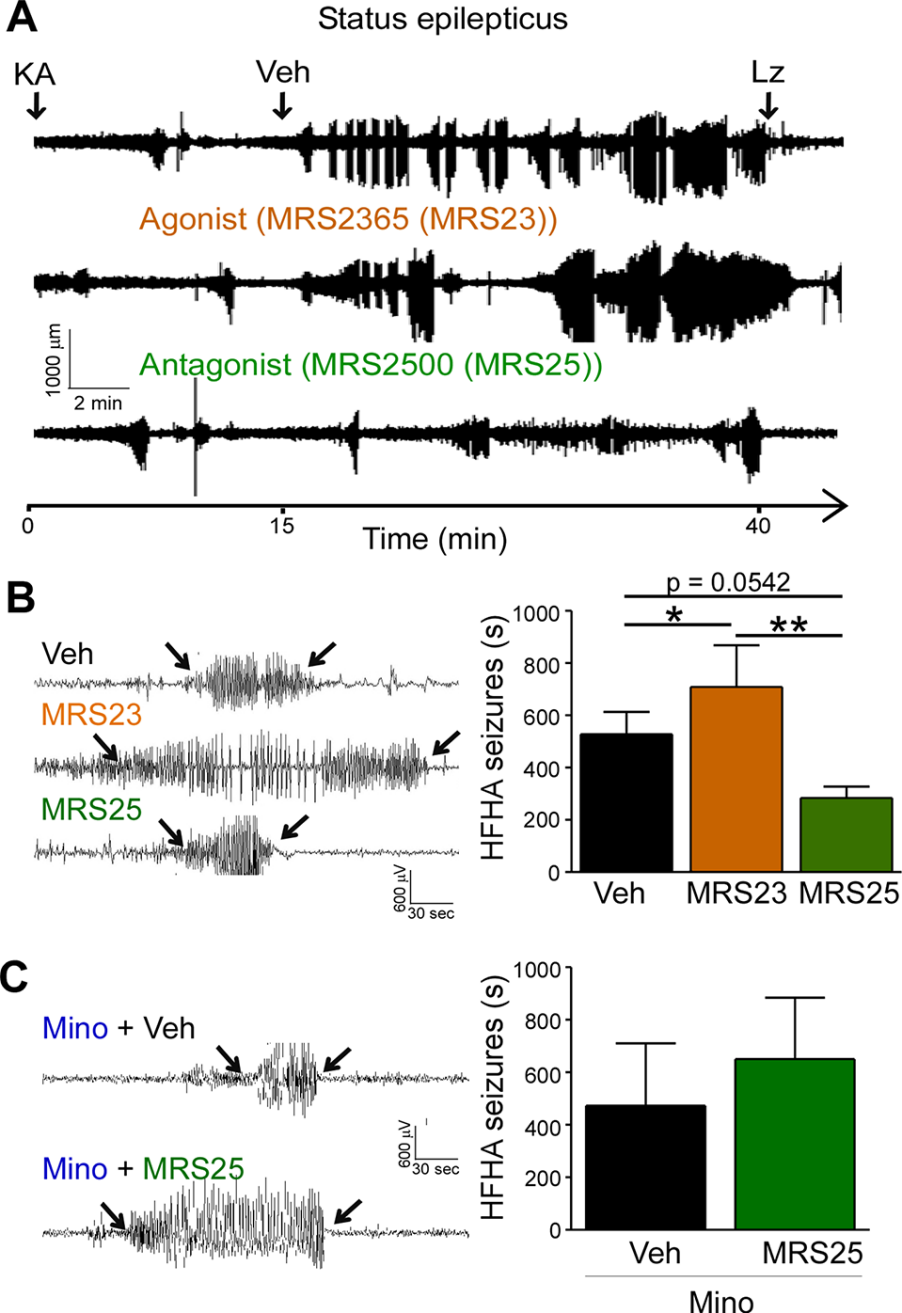
P2Y_1_ antagonism decreases high frequency high amplitude spiking during status epilepticus. **(A)** Representative EEG traces recorded from the cortex from the time-point of intraamygdala kainic acid (KA) injection until 60 min post-lorazepam (Lz) of mice treated with vehicle (Veh), P2Y_1_ agonist MRS2365 (MRS23), and P2Y_1_ antagonist MRS2500 (MRS25). Treatment with P2Y_1_-targeting drugs was administered 15 min post-intraamygdala KA injection *via* i.c.v. Lorazepam (Lz) was administered 40 min following KA injection *via* i.p. **(B)** Representative EEG traces showing examples of high frequency and high amplitude (HFHA) spiking taken during the 30 min recording period following drug treatment (see arrows). Mice treated with the P2Y_1_ agonist MRS23 showed an increase in the duration of HFHA spiking while mice treated with the P2Y_1_ antagonists MRS25 showed a decrease in the duration of HFHA spiking during status epilepticus (n = 7 Veh, 9 MRS23, and 8 MRS25). **(C)** Representative EEG traces and graph showing slightly increased duration of HFHA spiking during status epilepticus in mice treated with minocycline and P2Y_1_ antagonists MRS25 (n = 4 per group). *p < 0.05, **p < 0.01.

High frequency high amplitude (HFHA) spiking during status epilepticus has been shown to correlate with cell death in the intraamygdala KA mouse model (Araki et al., 2002). We have previously shown that treatment with the P2Y_1_ agonists MRS2365 (MRS23) during status epilepticus increased total seizure power and treatment with the P2Y_1_ antagonists MRS2500 (MRS25) reduced total seizure power (Alves et al., 2019). To determine whether targeting of P2Y_1_ also impacts on HFHA spiking and thereby potentially on seizure-induced neurodegeneration, we re-analyzed EEG traces and quantified HFHA spiking in mice subjected to intraamygdala KA and treated with the P2Y_1_ agonist MRS23 or P2Y_1_ antagonist MRS25 15 min following the induction of status epilepticus (Alves et al., 2019). This revealed that mice treated with the P2Y_1_ agonist MRS23 displayed a significant increase in HFHA spiking when compared to vehicle-treated mice (**Figures 3A, B**). No significant effect could be observed in mice treated with the P2Y_1_ antagonist MRS25 when compared to vehicle-injected mice, although MRS25-treated mice showed a ~40% reduction in HFHA spiking when compared to control (Veh (527.7 ± 85.61 s) vs. MRS25 (317.7 ± 38.57 s), *p* = 0.0542) (**Figures 3A, B**). To test whether effects of P2Y_1_ are mediated *via* inflammation, mice were treated with the broad-spectrum anti-inflammatory drug minocycline (Abraham et al., 2012; Alves et al., 2019). In line with anti-convulsive effects of P2Y_1_ antagonism being mediated *via* inflammation, mice pre-treated with minocycline and injected with the P2Y_1_ antagonist MRS25 showed no seizure reduction when compared to vehicle-injected mice subjected to intraamygdala KA status epilepticus and pre-treated with minocycline (**Figure 3C**).

In summary, P2Y_1_ activation contributes to HFHA spiking during status epilepticus possibly mediated *via* driving pro-inflammatory processes in the brain.

### P2Y_1_ Antagonism Protects the Cortex From Seizure-Induced Neurodegeneration

**Figure 4:**
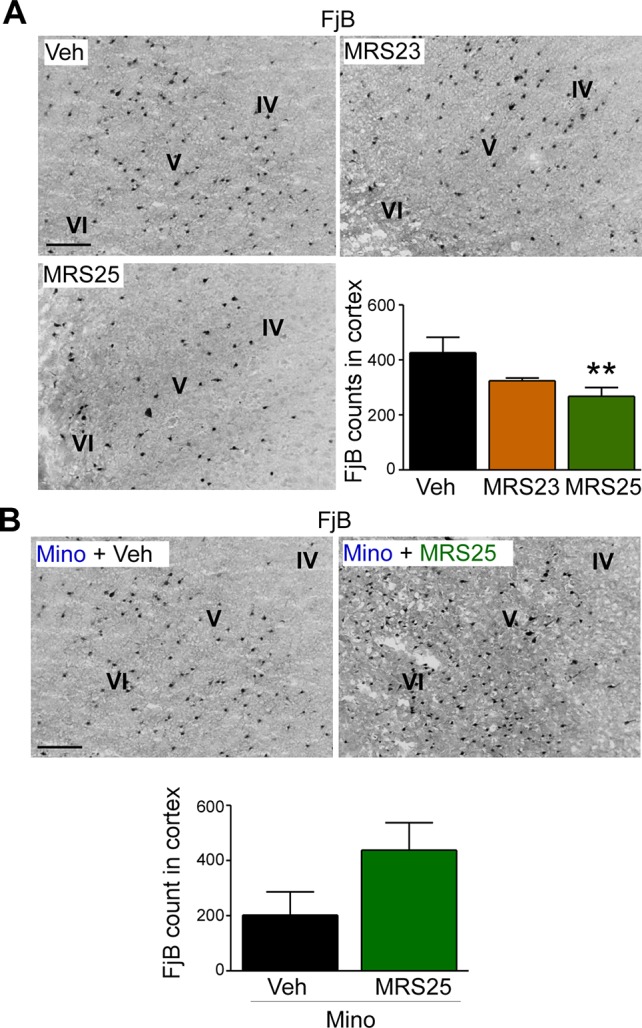
Decreased status epilepticus-induced cortical damage *via* P2Y_1_ antagonism. **(A)** Representative images (20× lens) and corresponding graph showing a decrease in neuronal damage in the cortex 24 h post-status epilepticus in mice treated with the P2Y_1_ antagonist MRS25 (n = 7 Veh, 9 MRS23, and 8 MRS25). **(B)** Representative images (20x lens) and corresponding graph showing slightly more Fluoro-Jade B (FjB)-positive cells in ipsilateral cortex 24 h post-status epilepticus in mice treated with both minocycline and the P2Y1 antagonist MRS25 (n = 4/group). **p < 0.01.

Several studies have reported P2Y_1_ antagonism to be protective against seizure-induced neurodegeneration in the hippocampus (Simoes et al., 2018; Alves et al., 2019). To determine whether P2Y_1_ antagonism also protects against cortical cell death, brain tissue was stained with the neurodegeneration marker FjB and cell death quantified in the cortex as before (Jimenez-Pacheco et al., 2013). While no significant effect on cortical neurodegeneration could be observed in mice treated with the P2Y_1_ agonist MRS23 (**Figure 4A**), treatment with the P2Y_1_ antagonist MRS25 reduced significantly neurodegeneration in the cortex (**Figure 4A**). Conversely, mice pre-treated with minocycline and then treated with the P2Y_1_ antagonist MRS25 during status epilepticus showed no difference in neurodegeneration in the cortex when compared to minocycline pre-treated vehicle-injected mice subjected to status epilepticus (**Figure 4B**).

In conclusion, our result suggests that P2Y_1_ antagonism not only reduces seizure severity during status epilepticus, but also protects the brain from damage including cortical tissue.

## Discussion

In the present study, by using a unilateral mouse model of status epilepticus, we report an overall increase in the expression of the P2Y receptor family in the cortex following status epilepticus and that P2Y_1_ antagonism reduces harmful HFHA polyspiking during status epilepticus and protects the cortex from seizure-induced neurodegeneration. Finally, we show that the observed neuroprotective effect provided by P2Y_1_ antagonism is mediated, at least in part, *via* reducing inflammation in the brain. Therefore, our study extends previous data demonstrating a detrimental role of P2Y_1_ activation during status epilepticus and further suggests that targeting of P2Y_1_ may represent a novel therapeutic avenue to treat patients with drug-refractory status epilepticus.

While there was little interest in P2Y receptors as potential drug-targets to treat status epilepticus or epilepsy, this has changed significantly over the past years with emerging evidence suggesting a causal role of this receptor family not only during seizure generation, but also in the development of epilepsy (Alves et al., 2018). Recent data published by us has demonstrated a distinct expression profile of the P2Y receptor family in the hippocampus following status epilepticus in both animal models and temporal lobe epilepsy patients (Alves et al., 2017). We have now extended these data analyzing the cortex, a structure in which status epilepticus also induces neuronal death (Curia et al., 2008; Mouri et al., 2008). Here we show that status epilepticus leads to an up-regulation of P2Y_1_, P2Y_2_, P2Y_4_, and P2Y_6_ in the cortex, with the P2Y_1_ and P2Y_4_ receptors showing the strongest increase. These results are in line with a former study where we have shown an upregulation of the Gq-binding P2Y receptors in the hippocampus post-status epilepticus (Alves et al., 2017). The observed status epilepticus-induced decrease in P2Y_12_ expression in the hippocampus could, however, not be replicated in the cortex. Moreover, P2Y expression changes occurred at later time-points following status epilepticus in the cortex when compared to the hippocampus with the only exception being P2Y_4_ which was increased shortly following status epilepticus in both brain structures. We do not know what the reason for these discrepancies between different brain areas are; however, different brain structures are differently affected by seizures during status epilepticus with seizures first occurring in the hippocampus when compared to the cortex (Engel et al., 2017). Differences in cell populations between cortex and hippocampus may further contribute to differences observed in the expression profile between both brain structures.

Indirect evidence suggesting a functional role for P2Y_1_ and P2Y_4_, P2Y receptors undergoing the strongest increase in their expression following status epilepticus, stems from a study showing that treatment with ADP, main endogenous P2Y_1_ agonist, increases seizure severity during status epilepticus and treatment with UTP, main endogenous agonists of P2Y_4_, decrease seizure severity (Alves et al., 2017). In line with this, P2Y_1_-targeting has been repeatedly shown to protect against status epilepticus using different experimental models of seizures and epilepsy (Alvarez-Ferradas et al., 2015; Nikolic et al., 2018; Simoes et al., 2018; Alves et al., 2019). However, in contrast to UTP-binding receptors being anticonvulsive, a more recent study using a rat model of KA-induced acute seizures has shown that blocking P2Y_4_ reduces seizure severity during status epilepticus (Zhang et al., 2019). Future studies using different mouse models of status epilepticus and different treatment regimens (pre-treatment vs. post-treatment) will have to clarify whether these observed effects of targeting P2Y_4_ are model- and/or treatment-specific. Nonetheless, the increased expression of both receptor subtypes following status epilepticus and during epilepsy suggests drugs targeting these receptors as possible therapeutic approaches for both drug-refractory status epilepticus and epilepsy.

Previously we have shown that in the mouse hippocampus P2Y_1_ is expressed in neurons under normal physiological condition and, following status epilepticus, is also detected on microglia (Alves et al., 2019). In the cortex, P2Y_1_ receptor expression was mainly detected on cortical neurons during physiological control conditions and post-status epilepticus, although at somewhat lower levels when compared to the hippocampus (Alves et al., 2019). In cortical microglia, P2Y_1_ expression was, however, as observed before in the hippocampus, almost undetectable during control conditions and strongly increased 24 h following status epilepticus (De Simone et al., 2010; Alves et al., 2019). Although P2Y_1_ has been described to be expressed on astrocytes under different pathological conditions, such as oxidative stress (Shinozaki et al., 2006; Fujita et al., 2009), ischemia (Zheng et al., 2013), and in patients with cortical dysplasia (Sukigara et al., 2014), no expression of P2Y_1_ was detected on astrocytes in the cortex in our status epilepticus mouse model. This is in agreement with our previous study showing absence of P2Y_1_ on astrocytes in the hippocampus post-status epilepticus (Alves et al., 2019). We included in our study a staining performed in the P2Y_1_ KO mouse as a negative control, demonstrating the specificity of the P2Y_1_ antibody. P2Y_1_ expression on cortical astrocytes may also be below the detection range of our antibody-based detection methods and electrophysiological techniques may be required. However, regardless whether P2Y_1_ is present on astrocytes or not, our results show that P2Y_1_ is also in the cortex strongly upregulated on microglia, suggesting P2Y_1_-driven microglia activation during status epilepticus not being restricted to the hippocampus.

Here, we also report that while treatment with P2Y_1_ agonists increases HFHA polyspiking during status epilepticus, P2Y_1_ antagonism reduces HFHA polyspiking. This is in line with previous findings showing a reduction in total severity of seizures during status epilepticus *via* P2Y_1_ antagonism (Alves et al., 2019). Suggesting this being neuroprotective, studies in the intraamygdala KA mouse model of status epilepticus have shown HFHA spiking to correlate with brain injury (Araki et al., 2002). In line with HFHA spiking causing neurodegeneration, mice treated with the P2Y_1_ antagonist MRS25 also showed less cell death in the cortex. However, despite the increase in HFHA polyspiking during status epilepticus caused by the P2Y_1_ agonist MRS23, this did not translate into more cell death in the cortex. The reason for this remains elusive; P2Y_1_ may have, however, effects independent on increasing hyperexcitability which impact on cell survival. In line with P2Y_1_ being anti-apoptotic, we have recently shown that P2Y_1_ overexpression protected against KA-induced neuronal death *in vitro* (Alves et al., 2019).

Finally, demonstrating P2Y_1_ contributing to seizure pathology at least in part *via* driving inflammation, anticonvulsive and neuroprotective effects conferred by P2Y_1_ antagonism were lost when mice were pre-treated with minocycline, which is in good agreement with our previous results examining the effects of P2Y_1_ signaling on the hippocampus (Alves et al., 2019). It has to be noted that minocycline treatment reduced cortical neurodegeneration following status epilepticus. It is, however, unlikely that this reduction in cell death had an impact on our results as both groups were pre-treated with minocycline. The cell-specific contribution of P2Y_1_ to the observed effects remains, however, to be elucidated. It is tempting to speculate that effects provided by P2Y_1_ antagonism are due to P2Y_1_ driving microglia activation. Indeed, our results show a dramatic increase in P2Y_1_ immunoreactivity on microglia post-status epilepticus and the broad-spectrum anti-inflammatory drug minocycline has been shown to act predominately on this glial cell type (Abraham et al., 2012; Alves et al., 2019). Importantly, a role for P2Y_1_ on microglia activation has been repeatedly demonstrated previously (Davalos et al., 2005; Farber and Kettenmann, 2006; De Simone et al., 2010) with studies showing P2Y_1_-mediated signaling on microglia to affect neurodegeneration during ischemia and traumatic brain injury (Shinozaki et al., 2017; Fukumoto et al., 2018). Moreover, microglia are the first cells to respond during brain inflammation and microglia also respond rapidly to acute neuronal hyperactivity during seizures *via* NMDA-type glutamate receptors (Davalos et al., 2005; Eyo et al., 2014). We cannot rule out, however, a contribution of other cell types such as neurons or astrocytes. To fully prove the cell-specific contribution to P2Y_1_-mediated effects, this would require the use of cell-specific P2Y_1_-deficient mice. Nevertheless, our results strongly suggest that the anticonvulsive and neuroprotective effects mediated *via* P2Y_1_ signaling are due to P2Y_1_ driving inflammatory processes.

In conclusion, our study extends previous data confirming anticonvulsive and neuroprotective properties of P2Y_1_ antagonism during status epilepticus, further suggesting P2Y_1_-based treatment as possible new therapy for drug-resistant status epilepticus.

## Data Availability Statement

All datasets generated for this study are included in the article.

## Ethics Statement

All animal studies were reviewed and approved by the Research Ethics Committee of the Royal College of Surgeons in Ireland.

## Author Contributions

MA carried out Western blotting and EEG analysis and wrote parts of the manuscript. JS performed immunohistochemistry and wrote parts of the manuscript. TE supervised study, carried out *in vivo* work, and wrote the manuscript.

## Funding

This work was supported by funding from the Health Research Board HRA-POR-2015-1243 and from Science Foundation Ireland [17/CDA/4708 and 16/RC/3948 (co-funded under the European Regional Development Fund and by FutureNeuro industry partners)].

## Conflict of Interest

Author JS was employed by FutureNeuro. The authors declare that this study received funding from FutureNeuro. The funder was not involved in the study design, collection, analysis, interpretation of data, the writing of this article or the decision to submit it for publication.

The remaining authors declare that the research was conducted in the absence of any commercial or financial relationships that could be construed as a potential conflict of interest.

The handling editor declared a past co-authorship with several of the authors MA, TE.
